# Developing an empirical model for spillover and emergence: Orsay virus host range in *Caenorhabditis*

**DOI:** 10.1098/rspb.2022.1165

**Published:** 2022-09-28

**Authors:** Clara L. Shaw, David A. Kennedy

**Affiliations:** Department of Biology, The Pennsylvania State University, University Park, PA 16802, USA

**Keywords:** host range, spillover, emergence, *Caenorhabditis*, Orsay virus, host jump

## Abstract

A lack of tractable experimental systems in which to test hypotheses about the ecological and evolutionary drivers of disease spillover and emergence has limited our understanding of these processes. Here we introduce a promising system: *Caenorhabditis* hosts and Orsay virus, a positive-sense single-stranded RNA virus that naturally infects *C. elegans*. We assayed species across the *Caenorhabditis* tree and found Orsay virus susceptibility in 21 of 84 wild strains belonging to 14 of 44 species. Confirming patterns documented in other systems, we detected effects of host phylogeny on susceptibility. We then tested whether susceptible strains were capable of transmitting Orsay virus by transplanting exposed hosts and determining whether they transmitted infection to conspecifics during serial passage. We found no evidence of transmission in 10 strains (virus undetectable after passaging in all replicates), evidence of low-level transmission in 5 strains (virus lost between passage 1 and 5 in at least one replicate) and evidence of sustained transmission in 6 strains (including all three experimental *C. elegans* strains) in at least one replicate. Transmission was strongly associated with viral amplification in exposed populations. Variation in Orsay virus susceptibility and transmission among *Caenorhabditis* strains suggests that the system could be powerful for studying spillover and emergence.

## 1. Introduction

Disease spillover and emergence can have catastrophic consequences for the health of humans and other species. For example, SARS-CoV-2 spilled over into human populations [1] and became pandemic, killing more than 6 million people when this study was published [2]. Moreover, the frequency of spillover events and the rate of new disease emergence has been increasing in the recent past [3], endowing urgency to the task of understanding drivers of spillover and the progression to emergence. Studies in wild systems with ongoing spillover have provided substantial insights into the spillover and emergence process [4–6], but experimental manipulation to test hypotheses in these systems can be impractical due to ethical and logistical concerns. Moreover, disease emergence is so rare that it typically can only be studied retrospectively. Therefore, it remains a challenge to understand what factors facilitate emergence and how evolution proceeds in emerging pathogens.

Spillover requires that pathogens have the opportunity and the ability to exploit a new host; emergence requires that this opportunity and ability persist through time [5,7]. Opportunity could occur if hosts share habitats or resources. Ability may arise through mutations or may pre-exist due to pathogen plasticity or host similarity. Studies of natural spillover and emergence events have identified characteristics of pathogens, hosts and their interactions that generally support the above. For example, pathogens that successfully spill over are likely to be RNA viruses with large host ranges [8,9]. Likewise, hosts with close phylogenetic relationships are more likely to share pathogens than more distantly related hosts [9–14]. In addition, geographical overlap between hosts is associated with sharing pathogens [12], meaning that changes in host population distributions that bring new species into contact could potentially promote spillover and emergence events [9,15–17].

Ecological factors (e.g. host densities, distributions, diversity, condition and behaviour) can promote or hinder spillover by modulating host exposure risk or host susceptibility [5,7]. Likewise, it is believed that ecological factors can promote or hinder emergence through the modulation of onward transmission in spillover hosts, which determines whether pathogens meet dead ends in novel hosts, transmit in stuttering chains, or adapt and persist [18–20]. Conclusively demonstrating the influence of ecological factors, however, requires experimental manipulation, and it has so far been difficult to perform such studies.

Experimental model systems have been essential for testing hypotheses about infectious disease biology [21–23]. Indeed, major discoveries in immunity, pathogenesis, and pathogen ecology and evolution come from model systems such as *Mus musculus* [24], *Drosophila melanogaster* [25], *Daphnia* species [21], *Arabadopsis thaliana* [26] and *Caenorhabditis elegans* [27]. However, few model systems exist to study the ecology and evolution of disease spillover and emergence, and the systems that do exist lack key features known to drive disease dynamics (e.g. host behaviour or transmission ecology). A perfect model system would have large host population sizes, naturally transmitting, fast-evolving pathogens (e.g. viruses), and multiple potential host species with variable susceptibility and transmission.

*Caenorhabditis* nematode species are appealing model host candidates. Indeed, *C. elegans* and various bacterial and microsporidian parasites are staples of evolutionary disease ecology [22,28]. Specifically, the trivial manipulation and sampling of laboratory host populations mean that population-level processes like disease transmission and evolution can be observed, and the tractable replication of large populations makes possible the observation of rare events such as spillover and emergence. However, until 2011, there were no known viruses of any nematodes including *C. elegans*. That changed with the discovery of Orsay virus [29].

Orsay virus, a natural gut pathogen of *C. elegans*, is a bipartite, positive-sense, single-stranded RNA (+ssRNA) virus that transmits readily in laboratory *C. elegans* populations through the fecal-oral route [29]. This virus is an appealing model pathogen candidate since +ssRNA viruses have high mutation rates [30] and typically evolve quickly [31]. Moreover, since Orsay virus transmits between hosts in the laboratory, this system allows transmission itself to evolve, a critical component of emergence [19] that cannot be readily studied in other animal laboratory systems of disease emergence. To develop *Caenorhabditis* hosts and Orsay virus as a system for studying spillover and emergence, it is necessary to know the extent to which the virus can infect and transmit in non-*elegans Caenorhabditis* species. So far, such exploration has been limited to one other species, *C. briggsae,* which was determined to be refractory to infection [29]. Notably, an ancestral virus likely crossed at least one host species boundary in the past since *C. briggsae* has been found to be susceptible to three related viruses [29,32–34].

To explore the suitability of the *Caenorhabditis*–Orsay virus system for studies of disease spillover and emergence, we first test a suite of *Caenorhabditis* species for susceptibility to Orsay virus, and then we test the extent to which susceptible host species can transmit the virus. We establish lower bounds for both susceptibility and transmission ability, and we test for effects of host phylogeny on these traits*.* Although host ranges of various pathogens have been studied by infection assays (e.g. [35–38]) or by sampling infected hosts from natural systems (e.g. [11,39]), these studies do not typically distinguish between dead-end infections, stuttering chains of transmission, and sustained transmission. We found that nematodes varied in both susceptibility to the virus and their ability to transmit it, affirming the promise of this system for future studies of spillover and emergence.

## 2. Methods

### (a) Susceptibility assays

We assayed the susceptibility of *Caenorhabditis* species to Orsay virus by measuring virus RNA in virus-exposed host populations using quantitative PCR (qPCR). We obtained 84 wild isolate strains belonging to 44 *Caenorhabditis* species (one to three strains per species) from the *Caenorhabditis* Genetics Center (CGC) and from Marie-Anne Félix (IBENS). We tested each strain for Orsay virus susceptibility using eight experimental blocks (table 1; electronic supplementary material, table S1). Species identities were confirmed by sequencing the small ribosomal subunit internal transcribed spacer ITS2 and/or by mating tests. For each *Caenorhabditis* strain, we initiated three replicate populations with five adult animals. For sexual species, we used five mated females, and for hermaphroditic species, we used five hermaphrodites. All populations were maintained on nematode growth medium (NGM) in 60 mm diameter plates with a lawn of bacterial food (lawns were seeded with 200 µl of *Escherichia coli* strain OP50 in Luria-Bertani (LB) broth and allowed to grow at room temperature for approximately 24 h [40]). We exposed populations to virus by pipetting 3 µl of Orsay virus filtrate, prepared as described in [38], onto the centre of the bacterial lawn. We determined the concentration of the filtrate to be 428.1 (95% CI: 173.4, 972.3) × the median tissue culture infectious dose (TCID50) per µl (electronic supplementary material, Information A) [41]. We maintained populations at 20°C until freshly starved (i.e. plates no longer had visible bacterial lawns). Depending on the strain, this took anywhere from 3 to 28 days (electronic supplementary material, table S1). While this meant that strains may have experienced variable numbers of generations, this method ensured that all the exposure virus was consumed. We collected nematodes from freshly starved plates by washing plates with 1800 µl of water and transferring suspended animals to 1.7 ml microcentrifuge tubes. We centrifuged tubes at 1000 × g for 1 min to pellet nematodes. We removed the supernatant down to 100 µl (including the pellet of nematodes) and ‘washed’ external virus from nematodes by adding 900 µl of water and removing it five times, centrifuging at 1000 × *g* for 1 min between each wash. After the five washes, we lysed the nematodes by transferring the nematode pellet along with 500 µl of water to 2 ml round-bottom snap cap tubes, adding approximately 100 µl of 0.5 mm silica beads and shaking in a TissueLyser II (Qiagen) for 2 min at a frequency of 30 shakes per second. We then removed debris with two centrifugation steps of 17 000 × *g* for 5 min, each time keeping the supernatant and discarding the pellet. Samples were stored at −80°C.

**Table 1.** Strains were assayed for susceptibility to Orsay virus with the number of replicates processed in each block. When strains were assayed in multiple blocks, replicate numbers are given in the respective order of the blocks. Strains were acquired from the CGC (University of Minnesota) and from Marie-Anne Felix (IBENS).

strain	species	block	number of replicates	strain	species	block	number of replicates
JU1199	*C. afra*	2	3	JU2613	*C. portoensis*	7	3
JU1198	*C. afra*	4	3	JU2745	*C. quiockensis*	2	3
JU1593	*C. afra*	7	3	MY28	*C. remanei*	2	3
NIC1040	*C. astrocarya*	3	1	PB206	*C. remanei*	6	3
QG704	*C. becei*	2	3	JU1082	*C. remanei*	6	3
SB280	*C. brenneri*	1	3	JU1201	*C. sinica*	1	3
SB129	*C. brenneri*	6	3	JU4053	*C. sinica*	4	3
LKC28	*C. brenneri*	6	3	JU1202	*C. sinica*	6	3
JU1038	*C. briggsae*	1,2,3^a^	3,3,3	JU2203	*C.* sp. 8	5	2
EG4181	*C. briggsae*	6	3	QG555	*C.* sp. 24	3	3
ED3083	*C. briggsae*	6	3	JU2867	*C.* sp. 24	5,7	1,3
JU1426	*C. castelli*	3,7	3,3	JU2837	*C.* sp. 24	6	3
JU1333	*C. doughertyi*	1	3	ZF1092	*C.* sp. 25	3	3
JU1328	*C. doughertyi*	4	3	QX2263	*C.* sp. 27	1,3	2,3
JU1331	*C. doughertyi*	5	3	DF5152	*C.* sp. 30	3	3
DF5112	*C. drosophilae*	3	3	NIC1070	*C.* sp. 43	2	3
GXW1	*C. elegans*	6	3	JU4050	*C.* sp. 62	5	3
JU1401	*C. elegans*	6	3	JU4045	*C.* sp. 62	7	3
ED3042	*C. elegans*	6	3	JU4056	*C.* sp. 63	6	3
NIC113	*C. guadaloupensis*	1	3	JU4061	*C.* sp. 64	6	3
EG5716	*C. imperialis*	3	3	JU4087	*C.* sp. 65	4	3
JU1905	*C. imperialis*	7	3	JU4093	*C.* sp. 65	5	3
NKZ35^b^	*C. inopinata*	3	3	JU4092	*C.* sp. 65	5	3
QG122	*C. kamaaina*	2	3	JU4094	*C.* sp. 66	4	3
VX80	*C. latens*	1	3	JU4096	*C.* sp. 66	4	3
JU3325	*C. latens*	4	3	JU4088	*C.* sp. 66	4	3
JU724	*C. latens*	5,7	1,3	SB454	*C. sulstoni*	2	3
JU1857	*C. macrosperma*	2	3	JU2774	*C. tribulationis*	1	3
JU1865	*C. macrosperma*	5	3	JU2776	*C. tribulationis*	5	3
JU1853	*C. macrosperma*	7	3	JU2775	*C. tribulationis*	5	3
JU2884^c^	*C. monodelphis*	8	3	JU1373	*C. tropicalis*	1	3
JU1667^c^	*C. monodelphis*	8	3	JU1428	*C. tropicalis*	2	3
JU1325	*C. nigoni*	1,2,3	2,1,3	JU2469	*C. uteleia*	2	3
JU2617	*C. nigoni*	4	3	JU2458	*C. uteleia*	4	3
EG5268	*C. nigoni*	6	3	JU1968	*C. virilis*	3	3
JU1825	*C. nouraguensis*	1	3	JU2758	*C. virilis*	5	3
JU1833	*C. nouraguensis*	5	3	NIC564	*C. waitukubuli*	1	3
JU1854	*C. nouraguensis*	6	3	JU1873	*C. wallacei*	1	3
QG702	*C. panamensis*	2	3	EG6142	*C. yunquensis*	3	3
JU2770	*C. parvicauda*	7	3	JU2156	*C. zanzibari*	1	3
EG4788	*C. portoensis*	1	3	JU3236	*C. zanzibari*	6	3
JU3126	*C. portoensis*	5	3	JU2161	*C. zanzibari*	7	3

^a^JU1038 was included in the first three blocks as a type of negative control since a previous study found that *C. briggsae* was not susceptible. We discontinued this practice given the number of strains we needed to test.

^b^Strain NKZ35 was maintained at 23°C according to CGC recommendation.

^c^Populations were initiated with 12 juvenile animals due to challenges rearing animals with standard methods.

We used qPCR to measure viral RNA in these samples. Primers and probe were Forward: GTG GCT GTG CAT GAG TGA ATT T, Reverse: CGA TTT GCA GTG GCT TGC T, Probe: 6-FAM-ACT TGC TCA GTG GTC C-MGB. We performed 10 µl reactions composed of 1.12X qScript XLT One-Step RT-qPCR ToughMix (Quantabio), 200 nM each of forward and reverse primers and probe, and 2 µl of sample. Reaction conditions were 50°C (10 min), 95°C (1 min), followed by 40 cycles of 95°C (3 s), 60°C (30 s). Assays were run on a 7500 Fast Real-Time qPCR System (Thermo Fisher Scientific, Applied Biosystems). Cycle threshold (Ct) values were determined using the auto-baseline and auto-threshold functions of the 7500 Fast Real-Time software (Thermo Fisher Scientific, Applied Biosystems).

Each experimental block also contained five sets of controls and benchmarks (table 2). Control 1 was a negative control where *C. elegans* laboratory strain N2 was exposed to water instead of virus. Controls 2 and 3 were positive controls where *C. elegans* strains known to have moderate (N2) and high (JU1580) susceptibility were exposed (control 2, strain N2: mean(Ct) = 15.7, s.d.(Ct) = 2.0; control 3, strain JU1580: mean(Ct) = 12.7, s.d.(Ct) = 2.2). Benchmark 4 was used to determine a Ct threshold for overt infection (i.e. susceptibility); we added virus to OP50-seeded NGM plates without nematodes and treated them identically to our plates with exposed nematodes during extractions. Therefore, these plates were used to quantify the amount of exposure virus that remains after the washing and extraction procedure (benchmark 4: mean(Ct) = 38.4, s.d.(Ct) = 2.6). Benchmark 5 was used to quantify the maximum amount of virus that could be present without replication and thus to generate a highly conservative Ct threshold for infection; it was determined by diluting 3 µl of exposure virus into 497 µl water, which corresponds to the final volume of our extractions. Samples with more virus than benchmark 5 therefore give unequivocal evidence of virus amplification (benchmark 5: mean(Ct) = 22.0, s.d.(Ct) = 0.6). In practice, benchmark 5 is overly conservative as a threshold for determining infection because virus is expected to be washed away during the wash steps, extractions are likely to be less than 100% efficient, and the virus may degrade between exposure and extraction. We therefore used benchmark 4 and the within-strain standard deviation in Ct among plates to set a threshold for determining infection status based on Ct. We calculated variance in the Cts for each strain (with undetectable virus assigned a Ct of 40), found the mean variance and took the square root; the result (sqrt(var(Ct)) = 4.1) is equivalent to the standard deviation in Ct values within a strain. We set a threshold of one standard deviation more virus than the maximum amount of virus detected in benchmark 4 plates (Ct = 33.6), yielding a threshold of Ct < 29.5. Strains were considered susceptible if at least one replicate population had more virus than this threshold. Note that had we used benchmark 5 rather than benchmark 4 to determine infection status, only 4 of 21 strains would have changed susceptibility designation (JU2837, JU4056, JU4088 and JU4096). To confirm that virus was replicating within novel hosts deemed to be susceptible, we measured virus levels over time in three of our susceptible, novel host strains (electronic supplementary material, Information B, figure B1).

**Table 2.** Description of controls and benchmarks included in triplicate in each of the eight blocks of the susceptibility assays.

control/benchmark	description	type
1	laboratory *C. elegans* strain N2 exposed to 3 µl of water	negative control
2	laboratory *C. elegans* strain N2 exposed to 3 µl of Orsay virus filtrate	positive control
3	highly susceptible *C. elegans* strain JU1580 exposed to 3 µl of Orsay virus filtrate	positive control
4	3 µl of Orsay virus filtrate pipetted on the centre of bacterial lawn with no nematodes	threshold^a^
5	3 µl of Orsay virus filtrate added directly to 497 µl of water, yielding the final extraction volume for experimental samples	threshold^b^

^a^The purpose of this benchmark was to quantify exposure virus remaining in samples after five rounds of washing.

^b^The purpose of this benchmark was to quantify the maximum amount of virus that could be present in the absence of viral replication (i.e. total amount of virus added to each plate).

### (b) Transmission assays

We conducted transmission assays for all strains where at least one replicate population was determined to be infected in our susceptibility assay. First, three replicate populations were initiated as above and exposed to 3 µl of virus filtrate. At the same time, we initiated three replicate positive control populations of *C. elegans* laboratory strain N2 exposed to 3 µl of virus filtrate and three replicate negative control populations of strain N2 exposed to 3 µl of water. When populations were recently starved, 20 adult nematodes (mated females for sexual species or hermaphrodites for hermaphroditic species) were chosen at random and passaged to virus-free plates with fresh food (*E. coli* strain OP50 lawns prepared as above). Remaining animals were washed from the starved plates, virus was extracted and viral RNA quantified via qPCR as above (electronic supplementary material, table S2). We passaged each replicate line five times, or until there was no detectable viral RNA by qPCR. Controls were passaged five times regardless of virus detection.

We assigned each passage line a transmission score of 0, 1, 2 or 3 based on detection of viral RNA through the passages. A value of 0 was assigned when viral RNA was not detected in the exposure population; a value of 1 was assigned when viral RNA was detected in the exposure population but not in the first passage population; a value of 2 was assigned when viral RNA was detected in the first passage population but became undetectable on or before the fifth passage population and a value of 3 was assigned when viral RNA was still detectable in the fifth passage population.

### (c) Statistical analysis

We quantified phylogenetic relationships among nematode species using data from the most recent published phylogeny of *Caenorhabditis* [42]. We rooted the phylogeny with *Diploscapter pachys* as the outgroup and constrained the tree to be ultrametric (i.e. tips are all equidistant from the root—a requirement for our downstream analysis) using the ‘chronos’ function in the ‘ape’ package [43]. We selected a strict clock model since this method yielded the best ultrametric tree determined by the phi information criterion [44].

We then fit suites of Bayesian phylogenetic mixed effects models to the susceptibility and transmission data using the ‘MCMCglmm’ package [36,45,46] in R [47] (tables 3 and 4). Within each suite, models were compared using the deviance information criterion (DIC) to determine which model best explains the data (lowest DIC) and which model components are most important for describing patterns (see below) [48]. Best models according to DIC were used to draw additional conclusions about the significance of model components (see below). Data from controls and benchmarks were excluded from analyses of both the susceptibility and transmission data.

**Table 3.** Models compared for analysis of susceptibility patterns. All models included an intercept. The random effect of species is retained in all models to avoid pseudo-replication. ‘phylo. dist.’ indicates the effect of phylogenetic distance from *C. elegans* whereas ‘pairwise phylo. dist.’ indicates the effect of phylogenetic distance between species pairs.

model	ΔDIC	DIC weight
suscep.∼**fixed =** phylo. dist., **random =** pairwise phylo. dist. + species	0	0.544
suscep.∼**fixed =** phylo. dist., **random =** species	1.731	0.229
suscep.∼**fixed =** **random =** pairwise phylo. dist. + species	2.370	0.166
suscep.∼**fixed = random =** species	4.368	0.061

**Table 4.** Models compared for analysis of transmission scores. All models included an intercept. Random effects of species and strain are retained in all models to avoid pseudo-replication. ‘Ct’ indicates viral amplification on primary exposure plates. ‘phylo.dist.’ indicates the effect of phylogenetic distance from *C. elegans* whereas ‘pairwise phylo. dist.’ indicates the effect of phylogenetic distance between species pairs.

model	ΔDIC	DIC weight
trans.∼**fixed =** Ct + phylo. dist., **random =** pairwise phylo. dist. + species + strain	0	0.275
trans.∼**fixed =** Ct + phylo. dist., **random =** species + strain	0.518	0.212
trans.∼**fixed =** Ct, **random =** pairwise phylo. dist + species + strain	0.633	0.200
trans.∼**fixed =** Ct, **random =** species + strain	0.908	0.174
trans.∼**fixed =** phylo. dist., **random =** pairwise phylo. dist. + species + strain	4.015	0.037
trans.∼**fixed =** phylo. dist., **random =** species + strain	4.166	0.034
trans.∼**fixed =** **random =** species + strain	4.205	0.034
trans.∼**fixed =** **random =** pairwise phylo. dist. + species + strain	4.205	0.034

Two model components were included or excluded to generate our suite of models for the susceptibility data (table 3): a fixed effect of phylogenetic distance from *C. elegans* (calculated for each species with the ‘cophenetic.phylo’ function in ‘ape’ [45,48]) and a random effect of the inverse relatedness matrix between species pairs (i.e. the inverse of the matrix that contains the distance from the root to the common ancestor of any two species, calculated by the function ‘inverseA’ within the package ‘MCMCglmm’ [45,49]). The inverse relatedness matrix (hereafter referred to as ‘phylogenetic distance between pairwise sets of species') accounts for variation explained by phylogenetic relationships assuming a Brownian model of evolution [49]. An additional random effect of species accounts for differences among species that are not explained by phylogeny and was included in all models. Since our susceptibility data are binomial, we fit them using logistic regression with a logit link. In practice, this was achieved by setting family to ‘multinomial2’.

Three model components were included or excluded to generate our suite of models for the transmission data (table 4). Our most complicated transmission model included the two phylogenetic factors described above as well as an additional fixed effect of viral amplification in the primary exposure population measured as Ct, which was determined to likely be important upon plotting our data during preliminary analyses. All transmission models also included a random effect of species to account for differences between species that are not explained by phylogeny and a random effect of strain to account for replication at the strain level (table 4). Our transmission data are treated as continuous, and we fit them using linear regression by setting family to ‘gaussian’.

We used the MCMCglmm default priors for fixed effects (normal distribution with mean = 0 and variance = 10^8^) and parameter expanded priors for random effects that result in scaled multi-variate F distributions with V = 1, nu = 1, alpha.mu = 0, alpha.V = 1000 [50]. Residuals were assigned inverse Wishart priors with V = 1 n = 0.002 [50]. We ran models for 100 000 000 iterations with a burn-in of 30 000 and thinning interval of 5000. We visualized traces to affirm convergence of MCMC chains and confirmed stationarity with the test ‘heidel.diag’ in the package ‘coda’ [51]. The handful of models that had not converged were rerun with more iterations and larger thinning intervals to achieve convergence.

We compared models using DIC to select the best model. For the best model, we report posterior means and central posterior density 95% credible intervals as well as MCMC *p*-values for the fixed effects. Because *p*-values cannot be obtained for random effects, we also report the *R*^2^ values (calculated as described in [52]) for all model components included in our best model. We additionally used DIC to describe the relative support of each model and to further understand the importance of model components [48]. We calculated DIC weights for each model, each model component and the phylogenetic components combined [53]. The DIC weight of a model, calculated as $e^{-\Delta DIC/2}/\sum_{j}e^{-\Delta DIC/2}$ where *j* is the set of all models, gives the relative support for each model. Similarly, the DIC weight of a model component, calculated as $\sum_{i}e^{-\Delta DIC/2}/\sum_{j}e^{-\Delta DIC/2}$ where *i* refers to the set of models that includes a given parameter and *j* is the set of all models, is the posterior probability that a given component is included in the ‘true’ model assuming the ‘true’ model has been designated. Thus, model components with DIC weights greater than 0.5 are more likely than not to be included in the ‘true’ model.

## 3. Results

### (a) Susceptibility assays

**Figure 1.**
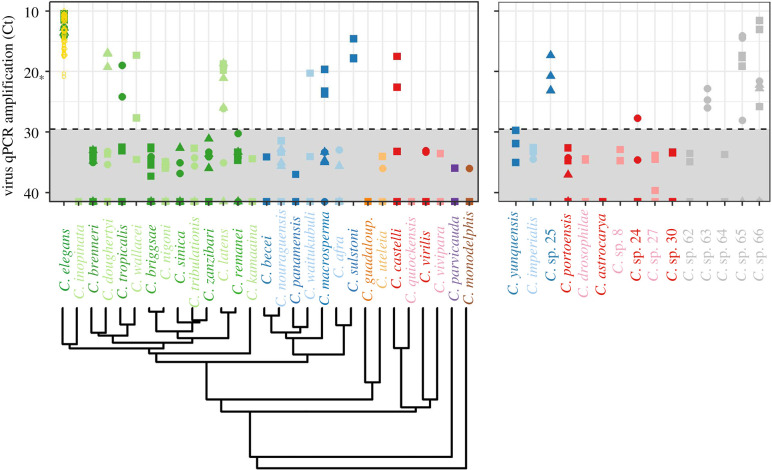
Species across the *Caenorhabditis* phylogeny are susceptible to Orsay virus (i.e. points above the infection determination cut-off (dashed line, see methods regarding ‘benchmark 4’). Note that smaller Ct values indicate more virus). The asterisk on the left side of the *y*-axis shows the Ct value from the ‘benchmark 5’ sample with the most detectable virus (table 2). The phylogeny (bottom left) is pruned from [42]. Many species currently have uncertain phylogenetic placement (right). Species for which a clade is hypothesized are colour-coded accordingly. These hypotheses were obtained from [51]. However, clades are unknown for *C*. sp. 62*, C*. sp. 63*, C.* sp. 64, *C.* sp. 65 and *C.* sp. 66. Shapes indicate different strains within a species; colours differentiate clades, but are otherwise only varied to aid visualization. Open gold circles and diamonds indicate Ct values for positive controls (‘control 2’ and ‘control 3’ plates, respectively; table 2). (Online version in colour.)

In our assays of host susceptibility to Orsay virus, we identified 21 susceptible *Caenorhabditis* strains of the 84 experimental strains tested (figure 1). These included three (non-control) strains of *C. elegans* (note that one of these strains, JU1401, had been previously documented to be susceptible [54,55]) and 18 strains belonging to 13 other species. In total, we found that Orsay virus is capable of infecting hosts from at least 14 of 44 *Caenorhabditis* species.

**Figure 2.**
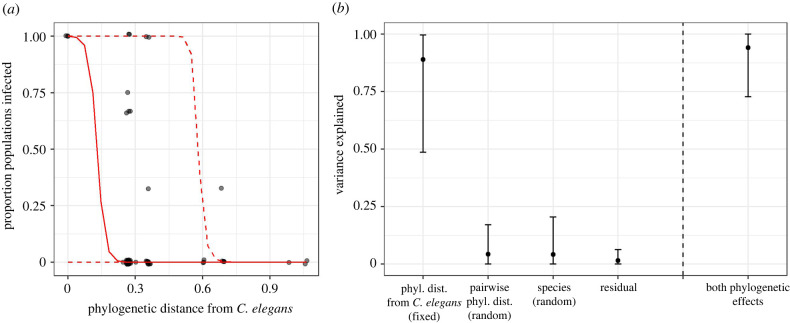
The best model for Orsay virus susceptibility included two phylogenetic components: a fixed effect of phylogenetic distance from the native host *C. elegans* and a random effect of phylogenetic distance between pairwise sets of species (table 3). (*a*) Slightly jittered points represent the proportion of exposed populations that became infected for a given strain plotted against the strains' phylogenetic distance from *C. elegans*. The solid red line shows the median model prediction. Dashed lines depict 95% credible intervals. (*b*) Points show the variance explained (*R*^2^) by each factor in the best model and error bars show 95% credible intervals [52]. (Online version in colour.)

Our statistical analysis uncovered the importance of host phylogeny in explaining differences in susceptibility. Our best model included both phylogenetic effects (table 3). In this best model, the fixed effect of phylogenetic distance from *C. elegans* was significant (pMCMC = 0.044, posterior mean: −81.56; CI: −272.31, −1.61; figure 2*a*). The importance of phylogenetic distance from *C. elegans* was also supported by the observation that susceptible strains were less well distributed across the phylogenetic tree than random (i.e. the mean distance from *C. elegans* of susceptible strains was 0.259 and ranged from 0 to 0.687, while the mean distance from *C. elegans* of all strains was 0.367 and ranged from 0 to 1.06). We also used *R*^2^ values from the best model and DIC weights calculated from the suite of models to further explore the importance of phylogenetic effects. Phylogenetic distance from *C. elegans* explained 89.0% (CI: 48.7%, 99.6%) of the variance in susceptibility (figure 2*b*) and had a DIC weight of 0.773. The random effect of pairwise phylogenetic distance explained 5.15% (CI: 0.0%, 22.0%) of the variance in susceptibility (figure 2*b*) and had a DIC weight of 0.710. Importantly, both phylogenetic effects together explained 94.1% (CI: 72.8%, 100%) of the variance (figure 2*b*), and models that included at least one of these phylogenetic effects had a weight of 0.939. Further, the model lacking either phylogenetic effect had a low DIC weight of 0.061, demonstrating additional support for the importance of phylogenetic effects [56]. The species-level random effect explained 4.2% (CI: 0.0%, 20.5%) of the variance in susceptibility (figure 2*b*); we were not able to compute a DIC weight for this component since it was included in all the susceptibility models.

### (b) Transmission assays

**Figure 3.**
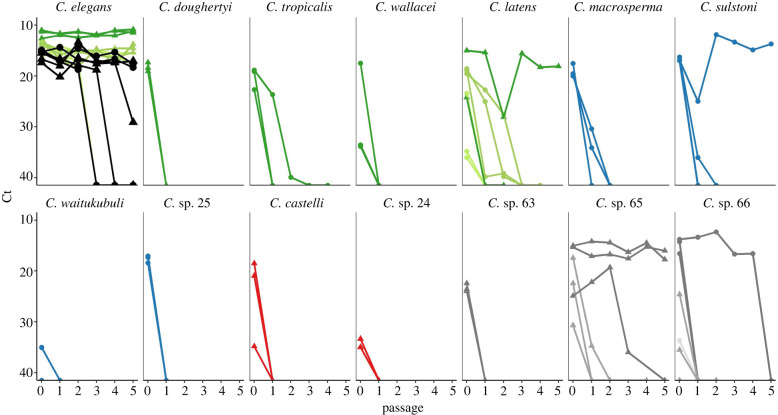
Orsay virus persisted to different extents when susceptible hosts were sequentially passaged to virus-free plates. ‘Passage 0’ denotes the primary exposure population. This experiment was carried out in two blocks indicated by shape (circle = block 1, triangle = block 2). N2 controls were present in both blocks, shown in black. Colours match colour-coded phylogeny in figure 1. Shades represent different strains within a species: *C. elegans* GXW1 (dark green), ED3042 (medium green), JU1401 (light green); *C. doughertyi* JU1331; *C. tropicalis* JU1428; *C. wallacei* JU1873; *C. latens* JU724 (dark green; one of the three replicate lines was removed from analysis due to bacterial contamination), VX80 (medium green), JU3325 (light green); *C. macrosperma* JU1857; *C. sulstoni* SB454; *C. waitukubuli* NIC564; *C*. sp. 25 ZF1092; *C. castelli* JU1426; *C.* sp. 24 JU2837; *C.* sp. 63 JU4056; *C.* sp. 65 JU4093 (dark grey), JU4087 (medium grey); *C.* sp. 66 JU4094 (dark grey), JU4088 (medium grey), JU4096 (light grey). (Online version in colour.)

We used the strains we identified to be susceptible in a subsequent transmission assay, which was completed in two blocks. Most replicates of *C. elegans* strains as well as positive control replicates (*C. elegans* strain N2) maintained high levels of virus through five passages (figure 2). However, virus was lost in one out of three control replicates in both blocks; in retrospect, this is unremarkable since the N2 strain used for controls is known to be less susceptible to Orsay virus than many other *C. elegans* strains [10,36,56]. Non-*elegans* strains did not transmit the virus as well in most cases. Virus was undetectable in the first passage population in all replicates of *C. doughertyi, C. wallacei, C. latens* strain JU3325, *C. waitukubuli, C*. sp. 25*, C. castelli, C.* sp. 24, *C.* sp. 63 and *C.* sp. 66 strains JU4088 and JU4096. Virus was also undetectable in the first passage population in one or two replicates of *C. tropicalis*, *C. latens* strain JU724, *C. macrosperma, C. sulstoni, C.* sp. 65 strain JU4087 and *C*. sp. 66 strain JU4094. Virus was maintained for 1 to 4 passages in at least one replicate of strains of *C. tropicalis, C. latens* strain VX80, *C. macrosperma, C. sulstoni, C*. sp. 65 strains JU4093 and JU4087*,* and *C.* sp. 66 strain JU4094. Virus was detectable through the 5^th^ passage in four non-*elegans* replicates belonging to three strains of different species: one replicate of *C. sulstoni* strain SB454, one replicate of *C. latens* strain JU724, and two replicates of *C.* sp. 65 strain JU4093 (figure 3).

The primary exposure populations (passage 0) in our transmission assay were treated nearly identically to populations in our susceptibility assay. As an internal control, we thus note high concordance between Ct measures in both assays (correlation coefficient = 0.85). In a separate experiment, we completed passages for additional replicates of two susceptible strains (*C. sulstoni* SB454 and *C. latens* VX80) for up to 12 passages, which yielded similar results to those in figure 3 demonstrating repeatability of our data (electronic supplementary material, Information B, figure B2).

**Figure 4.**
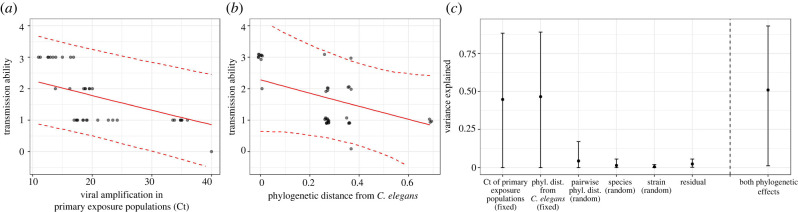
The best model for transmission ability included two fixed effects (viral amplification in primary exposure populations and phylogenetic distance from *C. elegans*) and three random effects (phylogenetic distance between pairwise sets of species, species and strain) (table 4). (*a*) Transmission ability was negatively associated with the Ct of primary exposure populations (i.e. positively associated with viral amplification) and (*b*) was negatively but non-significantly associated with phylogenetic distance from *C. elegans*. Note that points are jittered slightly. In (*a*) and (*b*), solid red lines depict the median effect size from the best model for how transmission ability declines with each fixed effect. Dashed lines represent central posterior density 95% credible intervals. (*c*) Variance explained by components in the best model [10,36,57]. (Online version in colour.)

As with the susceptibility data, we again identified factors associated with differences in transmission through model analysis. Our best model included a significant effect of viral amplification (Ct) in primary exposure populations (pMCMC = 0.009; posterior mean: −0.04; CI: −0.08, −0.01), a non-significant effect of phylogenetic distance from *C. elegans* (pMCMC = 0.132; posterior mean: −2.16; CI: −5.46, 0.95; figure 4*a,c*) and a random effect of phylogenetic distance between pairwise sets of species. Notably, the fixed effects were moderately correlated (correlation coefficient = 0.477).

Viral amplification in primary exposure populations explained 44.8% (CI: 0%, 88.3%; figure 4*b,c*) of the variation in transmission ability and had a DIC weight of 0.862. Phylogenetic distance from *C. elegans* explained 46.6% (CI: 0%, 89.0%) of the variation in transmission ability and had a DIC weight of 0.558, and pairwise phylogenetic distance between sets of species explained 4.3% (CI: 0%, 17.1%; figure 4*c*) of the variation in transmission and had a DIC weight of 0.546. Combined, the phylogenetic effects explained 50.9% (CI: 1.2%, 93.0%) of the variation in transmission ability, and models including at least one of the phylogenetic effects had a weight of 0.792. The *R*^2^ values and DIC weights indicate strong support for an effect of viral amplification in primary exposure populations and at least some support for each phylogenetic effect in explaining transmission ability despite the non-significant effect of phylogenetic distance from *C. elegans* in the best model. Interestingly, in the second-best model (table 4), which included phylogenetic distance from *C. elegans* and viral amplification in primary exposure populations but not the random effect of pairwise phylogenetic distance, phylogenetic distance from *C. elegans* was found to be marginally significantly associated with transmission ability (pMCMC = 0.083, posterior mean: −1.88, CI: −4.02, 0.35). Little of the variation in transmission ability was explained by species (*R*^2^ = 1.4%, CI: 0%, 5.6%) or strain (*R*^2^ = 0.5%, CI: 0%, 2.1%).

## 4. Discussion

In our study examining the host range of Orsay virus, we determined that at least 13 *Caenorhabditis* species in addition to *C. elegans* are susceptible to Orsay virus infection, but even within a species, strains may differ in susceptibility and transmission ability. Specifically, we found 21 susceptible *Caenorhabditis* strains (including three out of three *C. elegans* strains) out of 84 tested belonging to 44 species. When susceptible strains were assayed for transmission ability, 10 strains were dead-end hosts in all replicates and 6 strains (3 *C. elegans* strains, 1 *C. sulstoni* strain, 1 *C. latens* strain, and 1 *C.* sp. 65 strain) showed virus persistence for five passages in at least one replicate. The remaining five susceptible strains showed stuttering chains of transmission in at least one replicate. Our findings constitute lower bounds for the number of species and strains that are susceptible to Orsay virus and can transmit it; increased sampling of strains or increased replication could very well have identified more instances of susceptibility or transmission especially since these phenomena may be the result of stochastic ecological and evolutionary processes. Furthermore, we note that susceptibility and transmission findings are likely dependent on experimental conditions as we expect aspects of ecology such as dose and food quantity to impact spillover and emergence. Here, we found that susceptibility was associated with two phylogenetic effects: distance from *C. elegans* and phylogenetic distance between pairwise sets of species. Transmission ability was weakly associated with these phylogenetic effects according to analysis of DIC weights but strongly positively associated with viral amplification in primary exposure populations. Overall, we argue that the variation we observed among *Caenorhabditis* species and strains in susceptibility and transmission ability primes the *Caenorhabditis*–Orsay virus system to be valuable for experimental studies on the ecology and evolution of pathogen spillover and emergence.

Replicating findings from several other experimental studies of host range [58], we found evidence of phylogenetic effects on susceptibility. Host species more closely related to the native host *C. elegans* were more likely to be susceptible to infection, and closely related hosts had more similar susceptibilities regardless of their relationship to the native host. We expect that the importance of phylogenetic effects would only become more readily detectable if our unplaced *Caenorhabditis* species were placed on the phylogeny, since their lack of placement cost us statistical power. Importantly, we recovered an effect of phylogenetic distance from *C. elegans* even though few species are closely related to *C. elegans* (figures 1 and 2). A phylogenetic effect of susceptibility to related viruses (e.g. Santeuil, Le Blanc and Melnik [29,32–34]) might be even more readily detectable since the native host *C. briggsae* is a member of a clade with more closely related species.

We also tested for effects of phylogeny on transmission ability. Although patterns consistent with a phylogenetic effect on transmission have been identified [58], to the best of our knowledge, this has not been empirically documented. Our DIC analysis suggests that phylogenetic effects are important for transmission ability, but with weak statistical support likely resulting in part from the small number of hosts tested and their distribution across the phylogenetic tree. In addition, the moderate correlation between phylogenetic distance from *C. elegans* and our other focal fixed effect, viral amplification in primary exposure populations, may have made a phylogenetic distance effect more difficult to detect.

The use of DIC for model selection provided us with an objective tool for specifying a best model, and analysis of DIC weights allowed us to assess the relative importance of each factor included in our models. However, DIC is imperfect [59]. We elected to use it anyway because there was not a feasible alternative in our case [60]. We note that despite the shortcomings of DIC, we believe our conclusions from the DIC analysis are nevertheless robust. Notably, the average estimated effect for each factor was in the same direction across all models regardless of DIC score, and our *R*^2^ analysis provided conclusions consistent with our DIC weight analysis regarding the relative importance of our fixed and random effects.

Phylogenetic patterns in susceptibility may arise because closely related hosts likely have similar receptors, within-host environments and pathogen defenses [61,62]. Unfortunately, the receptor used by Orsay virus to enter host cells is currently unknown [61], and little is known about phylogenetic patterns in relevant within-host traits [61]. Exploring these traits may yield a more mechanistic understanding of determinants of Orsay virus competence. Notably, the important pathogen defense pathway RNA interference (RNAi) (i.e. where cellular machinery recognizes double-stranded RNA (dsRNA) and degrades corresponding viral RNA sequences) has been investigated across *Caenorhabditis* species [63]. This work uncovered phylogenetic patterns in the ability to respond to ingested dsRNA [64]. Importantly, most strains responded to some extent when dsRNA was injected [53], suggesting potential to mount an RNAi response to viral infection. Whether the nature and strength of the RNAi response is a mechanistic explanation for the patterns of susceptibility observed in our study remains to be explored formally, although we observed no obvious pattern between our data on susceptibility and the data on RNAi responses across species.

The strongest predictor of transmission ability in our study was viral amplification in primary exposure populations. We can imagine at least three reasons why amplification in primary exposure populations may matter for transmission. First, high levels of viral amplification may indicate that the virus was somewhat ‘pre-adapted’ and had the ability to infect and transmit among novel hosts without requiring any additional evolutionary changes [53,65]. Indeed, the correlation between viral amplification in primary exposure populations with phylogenetic distance from *C. elegans* is consistent with this idea. Second, if hosts can shed the virus, high levels of viral amplification may be indicative of higher shedding, meaning that hosts would encounter more virus, which could increase infection prevalence. If this was the case in our experiment, nematodes passaged from primary exposure populations with more viral amplification may have been more likely to have been infected. Third, larger virus populations may harbour more genetic variation, increasing opportunities for adaptive evolution that could maintain persistence of the virus in the spillover host. Indeed, evolutionary rescue theory has shown that larger populations are more likely to persist in comparison to smaller ones [65–67].

We also found substantial intra-species variation in susceptibility to Orsay virus. This result was somewhat expected because there is natural variation in susceptibility in the native host *C. elegans* [68–71]. Recent work has shown that the variation in *C. elegans* susceptibility can be partially attributed to genetic variation in two defense pathways: RNAi [71,72] and the intracellular pathogen response [72,73]. Future work may explore how genetic variation in these or other defense pathways influences Orsay virus susceptibility within novel host species. In addition to these known determinants of viral susceptibility in *C. elegans*, variation in gut physiology, behaviour, feeding rates, population density and demography may impact host susceptibility since these factors affect host–pathogen interactions in other systems (e.g. [74]).

Here we have documented spillover and transmission of Orsay virus in *Caenorhabditis* hosts. It is important to note, however, that the patterns we see with our susceptibility and transmission assays may not fully predict spillover and emergence patterns among *Caenorhabditis* hosts in the wild. Exposure risk is a key determinant of spillover and emergence [73], but in our experiments, we exposed all hosts equally. Orsay virus exposure risk for *Caenorhabditis* species in nature is unknown since we know little about the distributions of *Caenorhabditis* species and their viruses [74]. The two host species that have been most extensively studied in the wild, *C. elegans* and *C. briggsae,* do have overlapping distributions [75], but appear to be refractory to each other's viruses [76]. However, the fact that three viruses related to Orsay virus have been found in *C. briggsae* [29,32–34] suggests that at least one host jump has occurred in the past, since the viruses appear to be much more closely related [77] than *C. briggsae* and *C. elegans* [78].

The *Caenorhabditis*–Orsay virus system joins a small set of empirical systems suitable for studying spillover and emergence. Prior studies using other systems have yielded useful insights into these processes. For example, bacteria-phage systems have been used to show that the probability of virus emergence is highest when host populations contain intermediate combinations of native and novel hosts [79], that pathogen variation in reservoir hosts drives emergence in novel hosts [80,81] and that mutations that allow phages to infect novel hosts also constrain further host range expansion [81]. Plant–virus systems have been used to document the effects of host species on the fitness distribution of viral mutations [82], to determine the importance of dose, selection and viral replication for adaptation to resistant hosts [79], and to characterize how spillover can impact competition among host species [80,81]. *Drosophila*–virus systems have been used to show that viruses evolve in similar ways when passaged through closely related hosts [46] and to show that spillover dynamics can depend on temperature [82].

The *Caenorhabditis*–Orsay virus model can be uniquely useful for studying how ecology impacts spillover and emergence in animal systems since population characteristics like density, genetic variation and immunity can be readily manipulated and virus transmission occurs without intervention by a researcher. *Caenorhabditis* hosts have complex animal physiology, immune systems and behaviour, meaning that this system can be useful for revealing the importance of variation in these traits. In this study, we identified multiple susceptible spillover hosts that have variation in transmission ability. In the future, these hosts can be used not only to probe how ecology impacts spillover and emergence, but also to better understand how and why spillover and emergence patterns may differ across hosts.

## Data Availability

The code and data are accessible at https://github.com/clarashaw/Caenorhabditis-Orsay-virus-spillover. The data are provided in the electronic supplementary material [83].
